# Systematic Assessment of Blood-Borne MicroRNAs Highlights Molecular Profiles of Endurance Sport and Carbohydrate Uptake

**DOI:** 10.3390/cells8091045

**Published:** 2019-09-06

**Authors:** Fabian Kern, Nicole Ludwig, Christina Backes, Esther Maldener, Tobias Fehlmann, Artur Suleymanov, Eckart Meese, Anne Hecksteden, Andreas Keller, Tim Meyer

**Affiliations:** 1 Chair for Clinical Bioinformatics, Saarland University, 66123 Saarbrücken, Germany; fabian.kern@ccb.uni-saarland.de (F.K.); c.backes@mx.uni-saarland.de (C.B.); tobias.fehlmann@ccb.uni-saarland.de (T.F.); artur.suleymanov@uni-saarland.de (A.S.); 2 Department of Human Genetics, Saarland University Hospital, 66421 Homburg, Germany; n.ludwig@mx.uni-saarland.de (N.L.); Esther.Maldener@uks.eu (E.M.); Eckart.Meese@uks.eu (E.M.); 3 Center for Human and Molecular Biology, Saarland University Hospital, 66421 Homburg, Germany; 4 Department of Sports Medicine, Saarland University, 66123 Saarbrücken, Germany; a.hecksteden@mx.uni-saarland.de; 5 Center for Bioinformatics, Saarland University, 66123 Saarbrücken, Germany; 6 School of Medicine Office, Stanford University, Stanford, CA 94305, USA; 7 Department of Neurology and Neurological Sciences, Stanford University, Stanford, CA 94305, USA

**Keywords:** microRNA, physical exercising, circulating biomarker, homeostasis, randomized cross-over study, microarray, glucose nutrition, full-blood measurements, sncRNAs

## Abstract

Multiple studies endorsed the positive effect of regular exercise on mental and physical health. However, the molecular mechanisms underlying training-induced fitness in combination with personal life-style remain largely unexplored. Circulating biomarkers such as microRNAs (miRNAs) offer themselves for studying systemic and cellular changes since they can be collected from the bloodstream in a low-invasive manner. In *Homo sapiens* miRNAs are known to regulate a substantial number of protein-coding genes in a post-transcriptional manner and hence are of great interest to understand differential gene expression profiles, offering a cost-effective mechanism to study molecular training adaption, and connecting the dots from genomics to observed phenotypes. Here, we investigated molecular expression patterns of 2549 miRNAs in whole-blood samples from 23 healthy and untrained adult participants of a cross-over study, consisting of eight weeks of endurance training, with several sessions per week, followed by 8 weeks of washout and another 8 weeks of running, using microarrays. Participants were randomly assigned to one of the two study groups, one of which administered carbohydrates before each session in the first training period, and switching the treatment group for the second training period. During running sessions clinical parameters as heartbeat frequency were recorded. This information was extended with four measurements of maximum oxygen uptake (VO${}_{2}$ max) for each participant. We observed that multiple circulating miRNAs show expression changes after endurance training, leveraging the capability to separate the blood samples by training status. To this end, we demonstrate that most of the variance in miRNA expression can be explained by both common and known biological and technical factors. Our findings highlight six distinct clusters of miRNAs, each exhibiting an oscillating expression profile across the four study timepoints, that can effectively be utilized to predict phenotypic VO${}_{2}$ max levels. In addition, we identified miR-532-5p as a candidate marker to determine personal alterations in physical training performance on a case-by-case analysis taking the influence of a carbohydrate-rich nutrition into account. In literature, miR-532-5p is known as a common down-regulated miRNA in diabetes and obesity, possibly providing a molecular link between cellular homeostasis, personal fitness levels, and health in aging. We conclude that circulating miRNA expression can be altered due to regular endurance training, independent of the carbohydrate (CHO) availability in the training timeframe. Further validation studies are required to confirm the role of exercise-affected miRNAs and the extraordinary function of miR-532-5p in modulating the metabolic response to a high availability of glucose.

## 1. Introduction

The positive effects of sports activity on physical and mental health as well as the cardiovascular effects of training have been widely characterized [1,2,3]. In contrast, the current understanding of how genetic information is related to sports activity and how molecular processes are affected by exercise is still limited. Earlier studies showed that physical exercise has an impact on epigenetic factors, which are closely related to aging, such as DNA methylation levels, or the composition of histone modifications [4,5]. For example, Ludlow et al. demonstrated that exercising activates certain genes responsible for repairing DNA damage, in addition to the MAPK signaling pathway that ultimately contributes to telomere stability [6]. Also, expression levels of non-coding RNA transcripts in particular microRNAs (miRNAs) were found to be associated with exercising [7,8]. MiRNAs, typically 22 nucleotides in length, are loaded into proteins of the AGO family and orchestrate post-transcriptional gene regulation by targeting partially complementary sites in 3${}^{\prime }$ untranslated regions (UTRs) of messenger-RNAs (mRNAs) [9]. Circulating miRNAs, i.e., molecules carried by the bloodstream, constitute low-invasive biomarkers that can be extracted from whole-blood, serum, or plasma samples [10]. Previous work successfully utilized blood-borne miRNA profiles to identify adaptive molecular mechanisms as triggered by physical activities [11,12]. Adding yet another level of complexity, nutrition composition and personal dietary habits are known to influence systemic blood-glucose levels and training performance [13]. High blood-glucose availability specifically affects the drop in cellular ATP availability under physical stress conditions, which in turn determines the timing of AMP-activated protein kinase (AMPK) activation [14]. To this end, AMPK is of particular interest because it is thought not only to protect the cell from ATP shortage but also to function as the initial starting point in a signal cascade that governs the physiological adaption to regular physical exercising [15,16]. This motivates the question whether miRNA signatures being indicative of fitness levels after repeated endurance exercise, commonly measured via increasing values of maximal oxygen consumption VO${}_{2}$ max, exist and whether this is related to a varying abundance of glucose in blood [12].

Here, we analyzed 90 blood samples using microarrays to determine miRNA expression patterns from a randomized cross-over study to investigate the molecular relation between endurance sports and changes in VO${}_{2}$ max [17]. Since the repeated improvements of VO${}_{2}$ max across the study participants in the initial study were reported to be significant, we sought after possible relations between physiological training adjustment and circulating miRNA expression levels. Moreover, the influence of variations in carbohydrate-based nutrition on VO${}_{2}$ max levels was examined by searching for miRNAs that can differentiate between participants that show a positive training effect in combination with glucose uptake before each session and those that should refrain from any administration.

## 2. Results

### 2.1. Outline

**Figure 1:**
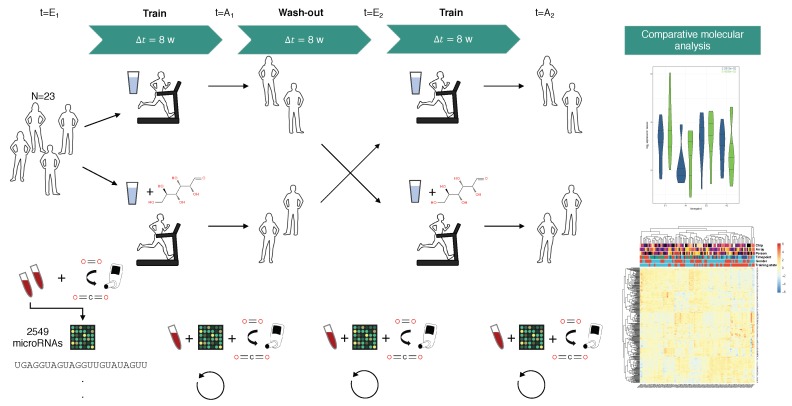
Overview on study design. Healthy and untrained participants were randomly assigned to any of two training groups, each performing of eight weeks of 4×45 min training, followed by a wash-out phase, again followed by eight weeks of endurance training. In the first period participants of one group orally administered glucose-solution 15 min before each running session, while participants of the second group administered carbohydrates in their second training period (cross-over). At four timepoints ($E_{1}$, $A_{1}$, $E_{2}$, and $A_{2}$) a blood-sample was taken and measured using complementary DNA (cDNA) microarrays probed with 2549 human microRNAs.

Our findings are based on a randomized cross-over study that consists of 23 participants, randomly split into two groups [17]. All participants were both healthy and untrained as indicated by a medical check including history taking, physical examination, resting, and exercise electrocardiography (ECG). The cohorts are $N_{1}=13$, $N_{2}=10$ consisting of $f_{N_{1}}=6$, $m_{N_{1}}=7$, and $f_{N_{2}}=4$ females, $m_{N_{2}}=6$ males, respectively. During study conduction participants were between 30 and 62 years old. Throughout the course of the study each participant completed eight continuous weeks with 4×45 min endurance training sessions per week, followed by a wash-out phase, and finally another eight weeks of training analogous to the first interval. Directly before and after each training interval, a blood-sample was taken from each participant, resulting in four timepoint measurements $E_{1}$, $A_{1}$, $E_{2}$, and $A_{2}$. In addition, during the first training interval participants that were assigned to the second group consumed 50 g glucose monohydrate dissolved in water, 15 min before each training session. Likewise, participants from the first group applied a carbohydrate solution before sessions of the second training phase (Figure 1, Supplementary Table S1). Furthermore, several anthropometric and fitness parameters such as the ventilatory threshold (VT1), maximum oxygen uptake (VO${}_{2}$ max), weight and body mass index (BMI), body fat levels, and maximal heart rate frequency were recorded (Supplementary Table S2) as well as a complete blood count (CBC) for each sample. While the significant improvements in VT and VO${}_{2}$ max after each training interval and in both study groups were reported earlier, we investigated changes among the other parameters as well [17]. Statistical tests revealed that the overall observed decrease in BMI and weight was not significant in any of the test arrangements, i.e., difference by study timepoint with and without treatment grouping. For body fat only the paired change in the non-CHO group for the first training interval was slightly significant (p≈0.044, paired Student’s *t*-test). Also, maximal heart frequencies declined during both training intervals, which was significant for the second period only (p≈0.046, Welch two-sample *t*-test). Blood-glucose levels were higher than baseline after each eight week training period and slightly raised for samples from the CHO treatment group as compared to non-CHO group, which did not yield statistical significance. Although red blood cell counts were observed to be reduced in both treatment groups following the exercising intervals, it only remained significant for the CHO treatment group (CHO: p≈0.017, Non-CHO: p≈0.151, Paired student’s *t*-test). All aggregated anthropometric and fitness parameters including test results are available from Supplementary Table S3. After preparation full-blood samples were hybridized using microarrays to measure the expression of 2549 human miRNAs from miRBase release v21 [18]. Overall, 90 of 92 samples could be measured successfully. Further, 307 miRNAs remained after removing those exhibiting either a low detection rate or a low-expression distribution across the samples. More details are provided in the methods section. While the average sample Spearman correlation for each sample is at or above 85%, an outlier sample could be identified that was subsequently excluded, leaving 89 samples for in-depth analysis (Supplementary Figure S1, top row and leftmost column). Also, no evident technical and biological batch effects could be found that pre-dominantly influenced the miRNA-sample matrix clustering (Supplementary Figure S2). However, there is a trend in both matrix clustering approaches for the samples to be grouped by the timepoint of blood extraction, notably not by each single timepoint individually but in a pairwise manner of pre-training ($E_{1}$, $E_{2}$) and post-training ($A_{1}$, $A_{2}$) timepoints, as indicated by the dichotomous variable *Training state*.

### 2.2. Physical Exercising Affects MicroRNA Expression

**Figure 2:**
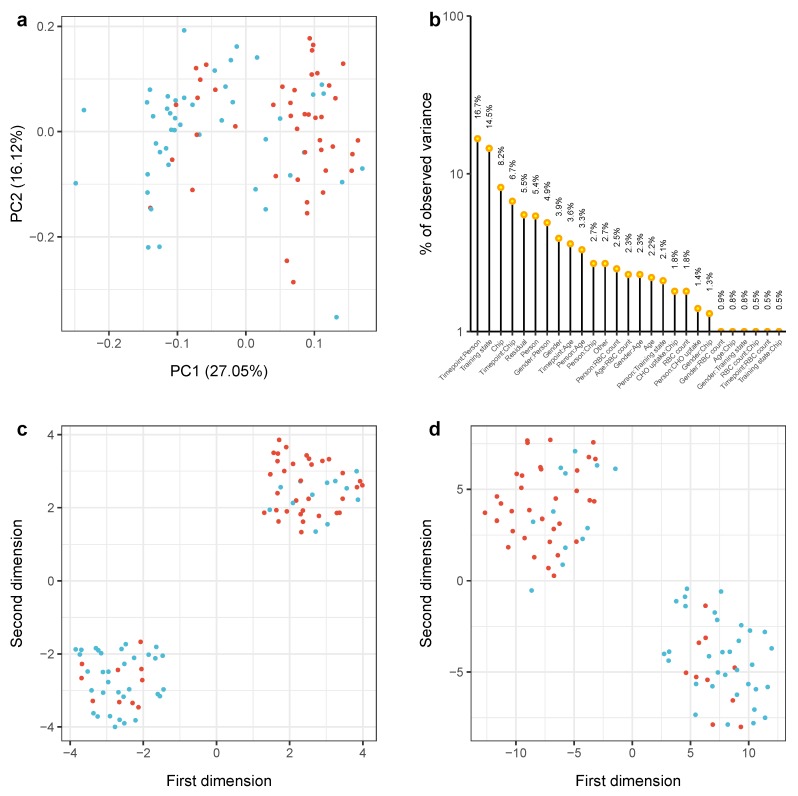
Analysis of variance and factors explaining it using measured miRNA expression data. (**a**) Sample distribution within the first two principal components obtained from principal component analysis (PCA) along with the percentage of variance explained in each dimension; (**b**) Results from principal variance component analysis (PVCA) showing estimates of variance in the expression data that can be explained with both known and unknown (hidden) sample annotation factors. Each bar corresponds to one factor, where mixed interactions between two variables are also possible and marked respectively by a colon; (**c**) two-dimensional uniform manifold approximation and projection (UMAP) embedding using the miRNA-sample matrix $X\in R^{90\times 307}$; (**d**) two-dimensional t-distributed stochastic neighbor embedding (t-SNE) embedding using the miRNA-sample matrix *X*. In each dimension reduction panel a single point corresponds to one sample that is colored according to the timepoint of blood extraction and relative to the training period, i.e., before (blue) or after (red) one of the training intervals.

To investigate whether endurance training is reflected by a molecular change of miRNA expression we analyzed the samples using both methods for dimension reduction and classical differential expression analysis. A two-dimensional embedding of the samples using principal component analysis (PCA) and the results for a corresponding batch variate assessment with principal variance component analysis (PVCA) are shown in Figure 2a,b [19,20]. In total, the first two principal components account for approximately 43% variance in the miRNA-sample matrix. As visible in Figure 2a samples measured before a training interval are enriched in the halfspace for which PC1≤0.02. Conversely, post-training samples are enriched in the halfspace PC1>0.02. Inspection of the sub-spaces spanned by the eigenvectors PC1 and PC2 did not yield an extreme distribution of prominent key-features but revealed a set of 65 miRNAs with a loading coefficient larger than 0.1 (Supplementary Table S4). Interestingly, the largest factor explaining the observed variance in miRNA expression is the interaction between timepoint and person connoted with each sample, suggesting that miRNAs exhibit a partially unique expression in whole-blood of healthy individuals, which is altered repeatedly by endurance training. Further, this observation is supported by the second most informative variable corresponding to whether a participant was untrained, i.e., the sample was taken before any training interval, or trained, i.e., the sample was taken after the eight-week training intervals. Technical factors seem to play a role as well, since the 3rd and 4th largest amount of variance can be explained using the information on which of the 17 microarray chips the sample was analyzed. Apparently, the gender seems to play a ubiquitous role as well, as it is estimated to explain approximately 13% of variance in total. Since it is known that endurance training influences the abundance of red blood cells (RBCs), which make up the largest proportion of cells within full-blood samples, we assessed whether a potential increase in RBC counts confounds the power of the variable “training state” in separating the samples. Given that we can estimate the variation of expression that is associated with technical and biological variables through PVCA, the total amount of variance which can be justified with a changing number of RBCs is close to 10% as illustrated by the different interaction variables in Figure 2b, suggesting it is not a driving factor. Remarkably, information whether the sample was taken after a training interval in which a participant administered carbohydrates as indicated by the surrogate variable “CHO uptake” substantiates only a minor fraction in our analysis. To overcome the inherent linear dependencies as uncovered through PCA, the more complex dimension reduction methods uniform manifold approximation and projection (UMAP) and t-distributed stochastic neighbor embedding (t-SNE) both further improved the separation of pre- and post-training samples, suggesting that non-linear effects play a role in our data set as well [21,22]. Because a similar number of the sample annotations disagree with the observed over-representation of “training state” in the two clusters, we investigated whether the samples belong to a specific set of participants that show a differential expression pattern, which turned out not to be the case. Indeed, both clusters show a significant enrichment according to a hypergeometric test with respect to the colored “training state” variable ($P\approx 4.3\times 10^{-6}$ for Cluster 1 located top-left, $P\approx 1.4\times 10^{-6}$ for Cluster 2 in bottom-right corner of Figure 2d).

**Figure 3:**
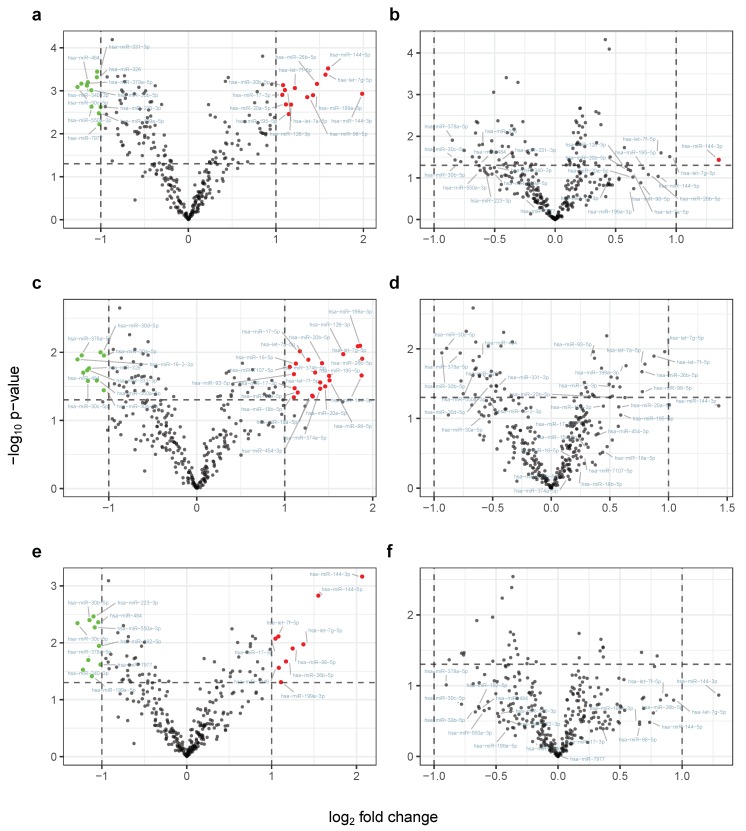
Volcano plots for six comparison setups using 307 microRNAs. The *x*-axis indicates log${}_{2}$ fold change, while the *y*-axis refers to the negative decade logarithm of *p*-values from student’s t-tests (unadjusted). Dashed horizontal lines indicate a *p*-value of 0.05, while dashed vertical lines indicate a log${}_{2}$ fold change of 2. (**a**) Pooling the samples from the two participant groups and using timepoints of the first training interval ($E_{1}$ and $A_{1}$); (**b**) pooled sample approach analogous to (**a**) but with timepoints from the second training period ($E_{2}$ and $A_{2}$); (**c**) analysis for timepoints like in (**a**) only using samples from the first treatment group; (**d**) analysis for timepoints like in (**b**) only using samples from the first treatment group; (**e**) analysis for timepoints like in (**a**) only using samples from the second treatment group; (**f**) Analysis for timepoints like in (**b**) only using samples from the second treatment group. MiRNAs that exceed both axis thresholds (dashed lines) are colored according to their direction of dys-regulation, i.e., red for up-regulation and green for down-regulation after training. For each row of panels, significantly de-regulated miRNAs on the left side (panels (**a**,**c**,**e**) are labelled and displayed on the corresponding right side (panels **b**,**d**,**f**).

Next, we assessed to which extent miRNAs are differentially expressed taking a pooled sample approach, once neglecting the CHO treatment groups, and once taking the different courses of training into account. Respective volcano plots for the different comparison setups are shown in Figure 3. After pooling samples from both groups for the first training interval ($E_{1}$, $A_{2}$), 11 miRNAs are significantly down-regulated after 8 weeks of endurance training with a log${}_{2}$ fold change ≤−1 and 13 miRNAs significantly increased in expression (Figure 3a; Table 1, Setup 1). Consulting the miRNA PCA-loadings for the two sets confirms their informative importance, each showing a larger mean of loadings than compared to the overall set of features ($mean_{down}\approx 0.099$, $mean_{up}\approx 0.099$, and $mean_{all}\approx 0.076$). MiRNA enrichment analysis on the set of down-regulated genes was accomplished with an online tool that computes which biochemical functional categories are significantly enriched in miRNA gene sets or lists (miEAA). The miEAA analysis revealed among others the protein-coding target gene APLN (p≈0.048, FDR<0.05) known to be associated with cardiovascular homeostasis and glucose metabolism [23,24]. For the group specific comparison, we found that 22 miRNAs are up-regulated and 10 are down-regulated in samples of participants that orally administered a glucose solution before each training session (Figure 3c; Table 1, Setup 2). In contrast, considering expression levels from participants that did not ingest a glucose solution 9 miRNAs are up-regulated while 10 show decreased expression (Figure 3e; Table 1, Setup 3). Despite the fact that not a single miRNA exhibits a contrary expression pattern between the two groups, which is either up-regulated in one group and down-regulated in the other, or the other way around, the observed set difference in de-regulated miRNAs remains significant ($p\approx 4.8\times 10^{-24}$, fisher’s exact test). Surprisingly, miR-144-3p is the only feature that is down-regulated using pooled samples of the second training interval (Figure 3b), denoting that no significant alterations for the CHO-treatment and group specific comparisons can be reported.

**Figure 4:**
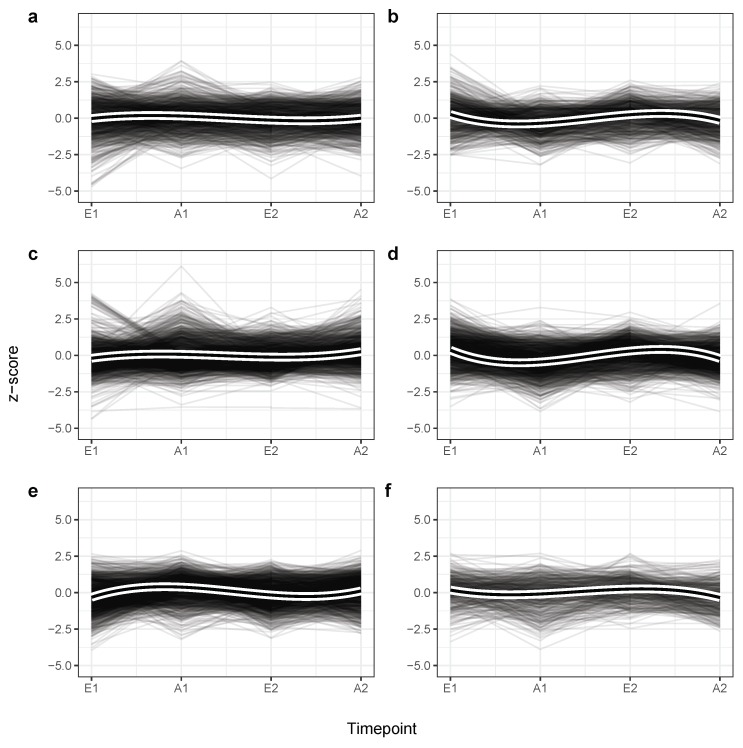
Distribution of Z-scores into six miRNA clusters $C_{1}$–$C_{6}$ corresponding to panel (**a**–**f**), along the four study timepoints using all samples. Each single grey line corresponds to one miRNA expression profile for one participant. Thick black lines display cluster-specific and smoothed curves of a cubic b-spline basis (b=3). The distinct clusters contain microRNAs that exhibit similar expression patterns over time. Although every cluster exhibits a wave-like expression pattern, miRNAs in $C_{1}$, $C_{3}$, $C_{5}$ show a tendency to be up-regulated after training (up, down, up) while those in $C_{2}$, $C_{4}$, and $C_{6}$ tend to be down-regulated after a period of endurance exercise (down, up, down).

Taking into account our findings about the differential expression of miRNAs we investigated if certain groups of miRNAs exist that exhibit similar expression patterns and whose expression is consistently changed after training. Using the full *z*-score scaled miRNA-sample expression matrix six miRNA expression clusters ($N_{C_{1}}=51$, $N_{C_{2}}=35$, $N_{C_{3}}=67$, $N_{C_{4}}=64$, $N_{C_{5}}=70$, $N_{C_{6}}=20$) were highlighted (Figure 4). The computed disjoint miRNA to cluster assignments are listed in Supplementary Table S5. Broadly speaking, the clusters can be separated into two larger expression classes, each showing a wave-like expression pattern with either a positive *z*-score peak first ($C_{1}$, $C_{3}$, and $C_{5}$) or a negative *z*-score peak first ($C_{2}$, $C_{4}$, and $C_{6}$). Notably, the direction of expression change is mostly maintained within in each cluster across the two training intervals, where the first peak, which corresponds to the first round of exercising, in general appears to be more prominent than the second, also reflected by the volcano plots in Figure 3. Nevertheless, differences between the clusters can also be observed, since $C_{1}$, $C_{3}$, and $C_{6}$ are much more attenuated than the remaining ones. These findings not only provide an explanation for the good separability of the samples with respect to trained and untrained participants, but propose that physical exercising may have diverse consequences on the molecular level and that a subset of the human miRNome is consistently affected by physical exercising.

### 2.3. MiRNA Expression Levels Correlate with Change in VO${}_{2}$ Max

Because our results indicate that miRNAs can be affected by endurance training, we put the analysis a step further and hypothesized that expression levels correlate with phenotypic changes in training performance observed through VO${}_{2}$ max. It is widely accepted that an improvement in VO${}_{2}$ max levels after endurance training denotes an improvement in the personal functional capacity. To this end, we computed the Spearman correlation between measurements VO${}_{2}$ max and miRNA expression values for each participant (Supplementary Figure S3). In the resulting correlation matrix three clusters of participants can be recognized, each showing an enrichment towards the group the participant were assigned to, i.e., CHO first and non-CHO second versus non-CHO first and CHO second training interval. Interestingly, several miRNAs show an opposing correlation towards VO${}_{2}$ max in-between the participant clusters, whereas some do not to show any robust correlation pattern.

**Figure 5:**
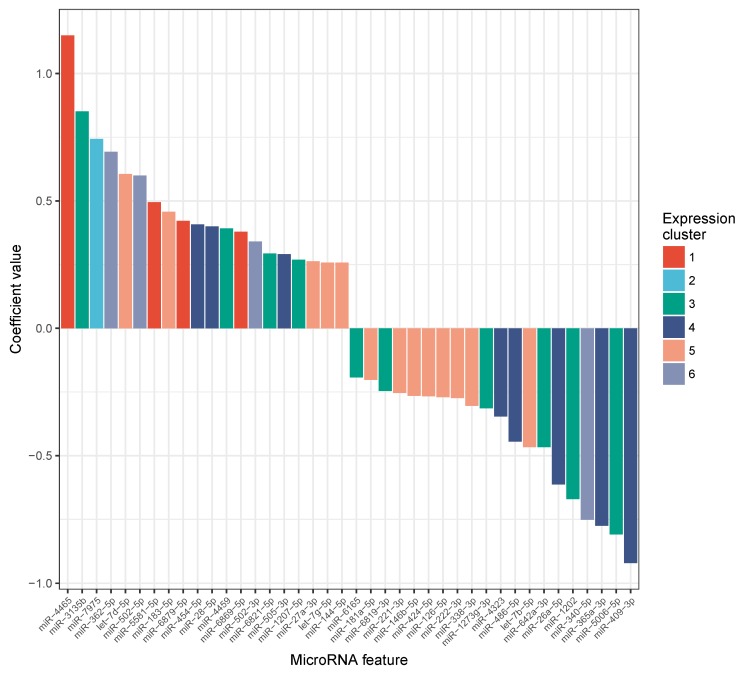
Top 20 positive and negative miRNA regression coefficients. Feature coefficients stem from the best linear model with respect to $R^{2}$ predicting the measured VO${}_{2}$ max values. In total 86 out of 307 miRNAs were assigned a coefficient unequal from zero. Each miRNA bar is colored according to its cluster identity ($C_{1}$–$C_{6}$) from Figure 4, highlighting a differential distribution of these clusters among the most important regression coefficients.

To pinpoint potential candidate miRNAs that exhibit a good correlation with the observed phenotype we performed linear regression using the miRNA expression values as features and the VO${}_{2}$ max values as dependent variable. Overall the model reached an estimated $R^{2}\approx 0.41$, suggesting that miRNAs partially explain the variance in VO${}_{2}$ max, yet questioning the existence of other factors that remain to be explored. In Figure 5 the distribution of the top 20 positive and top 20 negative feature coefficients from the best model selected with repeated cross-validation is shown. A link back to the expression groups reveals a dissimilar distribution of cluster identities between miRNAs with a different sign of coefficient. While positive coefficients are distributed among all clusters, cluster 1, 2, and 6 are mostly depleted in the top list of negative feature coefficients. This result affirms a putative correlation between exercise-induced, oscillating miRNA expression profiles and altered levels in VO${}_{2}$ max. In total 86 out of 307 miRNAs received a linear coefficient unequal from zero. Also, we report the absolute feature importance of each variable with a non-zero coefficient using the trained model (Supplementary Table S6). To check whether the miRNAs selected by our model are known to be associated with important molecular functions, we used miEAA to perform over-representation analysis. Among the pathways, organs, and biological functions enriched for the list of miRNAs having a non-zero feature importance we identified besides others; *Upregulated in male* ($P\approx 3.7\times 10^{-4}$, FDR<0.05), AMPK signaling ($P\approx 2.6\times 10^{-3}$, FDR<0.05, Wikipathways: WP1403), and Insulin signaling pathway ($P\approx 1.4\times 10^{-4}$, FDR<0.05, KEGG: hsa04910).

### 2.4. Candidate Marker MiR-532-5p Indicates Change in VO${}_{2}$ Max after Carbohydrate Uptake

**Figure 6:**
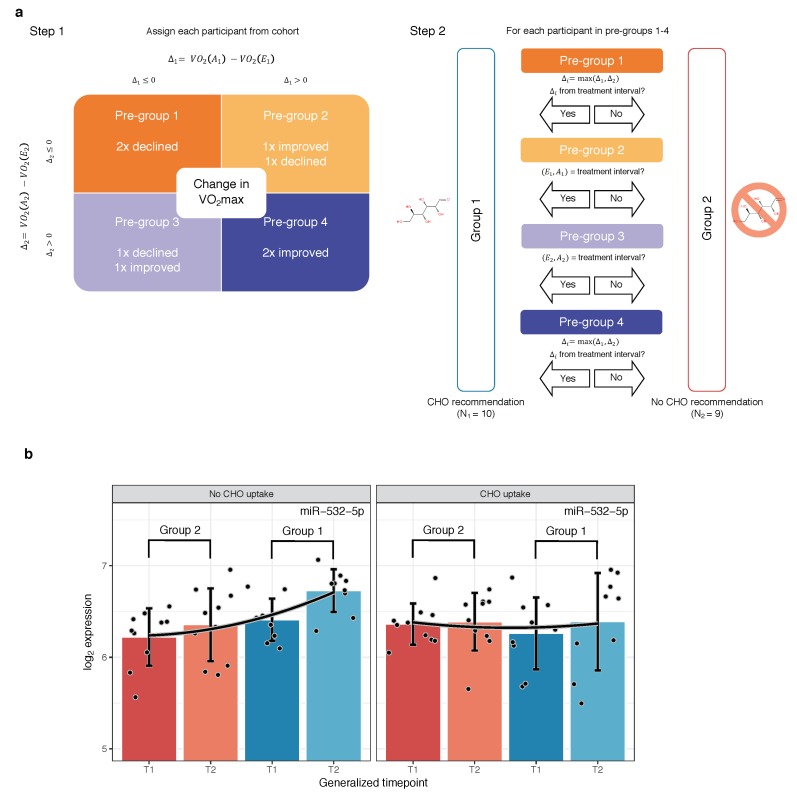
Assignment procedure conducted to find CHO recommendation groups and group-wise expression of candidate marker miR-532-5p. (**a**) Schematic workflow of devised procedure to classify each participant *p* into one of the two recommendation groups r(p)∈{0,1}. The decision process primarily depends on the personal change in VO${}_{2}$ max values during the first training period, $\Delta_{1}\;=\;VO_{2}\left(A_{1}\right)-VO_{2}\left(E_{1}\right)$ and $\Delta_{2}=VO_{2}\left(A_{2}\right)-VO_{2}\left(E_{2}\right)$ from the second training period. In a second step, participants are assigned to exactly one of the two recommendation groups based on which interval the better $\Delta VO_{2}$ occurred; (**b**) Paired barplots showing the expression of miR-532-5p across training period timepoints, i.e., $T1\in \{E_{1},E_{2}\}$ and $T2\in \{E_{2},A_{2}\}$, compared between the two CHO treatment recommendation groups, once using training periods without and once with an oral administration of glucose. Recommendation groups are highlighted by distinct colors where blue corresponds to a positive and red to a negative recommendation on glucose uptake. Each black point belongs to one sample in the distribution. Bar heights display the mean expression values and black error bars mean ± standard deviation. Smoothed quadratic b-splines (b=2) are drawn as black curves with a contrast margin.

Previous studies about the adaptive effects of a carbohydrate-rich nutrition exerted before training yielded conflicting conclusions, making it still unclear which impact a glucose-rich nutrition has on cardio–respiratory fitness [25,26,27]. In particular an impairment of cellular homeostasis conferred by AMPK under the variable influence of blood-glucose levels is known to control the training adaption process. Because healthy blood-glucose levels, the initial insulin response, and the upfollowing cellular insulin sensitivity can vary between individuals and with age, the conflicting results suggest the impact of carbohydrates is difficult to generalize. Therefore, we state the alternative hypothesis that this effect is highly individual, depending on many intra-personal factors such as sleep habits, age, gender, and genetic factors [28]. Hence, we asked whether the observed correlations between miRNA and VO${}_{2}$ max levels can be leveraged to separate the cohort in two recommendation groups, one that either showed a greater change in VO${}_{2}$ max in positive direction, i.e., greater improvement, or a smaller change in negative direction, i.e., less worsening under CHO treatment and the second group defined as the complement of group 1, namely participants for which the conditions apply under non-treatment periods. The grouping process is summarized and displayed in Figure 6a. Using the above outlined decision process we could separate the participants into two almost balanced groups (CHO recommended: $N_{1}=10$, CHO not recommended: $N_{1}=9$). Consequently, we assessed the miRNA expression levels for each timepoint, additionally taking into account the two recommendation groups. Manual inspection of 307 distributions highlighted a differential expression pattern of miR-532-5p, shown in Figure 6b and Table 2. In general, miR-532-5p is clearly expressed above background and potentially is affected by endurance training since both mean expression and standard deviation are higher for samples measured after any of the two training periods, independent of the recommendation assignments (Table 2, μ and σ increase row-wise and per-group from left to right). First, the difference in pre-training samples for which no carbohydrates were administered was not significant between the two groups (P≈0.15, Welch two sample t-test for $T_{1}$ dark red vs. $T_{1}$ dark blue, Figure 6b, left, Table 2; first row and first column), while the post-training difference turns out to be significant (P≈0.024, Welch two sample t-test for $T_{2}$ light red vs. $T_{2}$ light blue, Figure 6b, left; Table 2, first row and second column). Surprisingly, when repeating the analysis for the same recommendation groups but taking only the CHO treatment intervals into account, the differences vanish. Not only the pre-training difference remains to be insignificant (P≈0.51, Welch two sample t-test for $T_{1}$ dark red vs. $T_{1}$ dark blue, Figure 6b, right; Table 2, second row and first column) but also the post-training difference gets insignificant (P≈0.99, Welch two sample t-test for $T_{2}$ light red vs. $T_{2}$ light blue, Figure 6b, right; Table 2, second row and second column). Considering the four p-values, miR-532-5p has significantly higher expression levels in individuals from recommendation group 1 than group 2, when measured without any treatment and no significant difference with treatment. Following candidate selection, we investigated how miR-532-5p is expressed in-between the recommendation groups closely in relation to observed levels of VO${}_{2}$ max. To this end, we propose to assess the potential of miR-532-5p as a molecular marker in order to judge a possible effect of CHO uptake and a subsequent response in training performance by only considering the non-treatment period for each participant. We observed not only that the Spearman correlation between log${}_{2}$ fold change of miR-532-5p and change in VO${}_{2}$ max turns sign (ρ≈−0.4 pos. recommendation, ρ≈0.26 neg. recommendation) but miR-532-5p being even more up-regulated in the positive recommendation group (Figure 6b, left). Also, according to our group assignment strategy the mean change in VO${}_{2}$ max is lower as compared to the negative recommendation group. Conversely, for samples from the individual treatment periods, the Spearman correlation for the first group switches accordingly, while for the second it approximately remains the same. Hence, our results suggest that exercised-induced up-regulation of miR-532-5p is associated with observed levels of VO${}_{2}$ max and that it has potential discriminatory power in separating individuals who might administer carbohydrates before training from those that should refrain from a carbohydrate-rich nutrition. As a result these findings denote that two blood-samples for one training interval, precluding a treatment, and for any participant could in principle be used to judge an expected change of VO${}_{2}$ max under glucose treatment by investigating the expression levels of miR-532-5p in whole-blood.

## 3. Discussion

The prospective role of miRNAs as circulating biomarkers in diagnostics, which can be sampled in a low-invasive manner, has been already described by numerous publications [29,30,31]. However, technical factors such as sampling protocols, sample preparation, RNA detection technology, and statistical data analysis need to be fixed in order to account for potential bias and to make studies comparable among each other [32,33]. Here, we analyzed four timepoint measurements of 23 participants from a random cross-over study to investigate the effect of endurance-training on blood miRNA expression in combination with a variation of the glucose availability during training sessions. We found both technical factors, e.g., the microarray CHIP and biological factors, e.g., gender to determine most of the observed variance in expression. Even though one cannot rule out all possible influences of bias, especially in small-scale studies, the stringent study design and rigorous data analysis suggests that multiple miRNAs are differentially expressed after 8 weeks of regular endurance training. Albeit this effect was observed clearly for the first training interval, it remains unclear for the second. Nevertheless, a trend that spans both periods is reflected within our dimension reduction analysis where two clearly distinguishable clusters of samples from each endurance training period with differing CHO supply prior to each session can be recognized. To the best of our knowledge it cannot be explained completely with any observed variable but most appropriately by the binary training state. Interestingly, the number of miRNAs with significantly changed expression dropped substantially after the second training period, an observation for which we propose carry-over effects to be the most probable reason, also due to the fact that participants were completely untrained at the beginning of the study [17]. Nevertheless, the oscillating expression patterns of the six miRNA clusters displayed in Figure 4 are still indicative of an effect that is likely to be induced by the exhaustive training sessions. These observations highlight the importance of properly designed cross-over studies to prevent misleading conclusions based on non-reproducible one-time effects. To provide a remedy, substantially longer wash-out periods, for example being twice as long as the treatment periods, can be recommended.

Further, we observed a correlation between molecular changes in miRNA expression and phenotypic changes in VO${}_{2}$ max. Even though our miRNA enrichment analysis showed significantly associated biological categories such as the Insulin signaling pathway, the set of important miRNAs was rather large, possibly because some features are correlated among each other, motivating the need for further validation studies to increase specificity of the candidate set. While this result might also suggest an improved insulin sensitivity caused by a repetitive administration of glucose, our statistical results did not confirm any significance for either the glycemic profile or the differences in weight across the timepoints and order-of-treatment groups. Also, we put forward that expression differences observed between samples of different participants constitute a large proportion of variance in the data making it difficult to generalize the interrelations due to the fact that healthy samples from the same donor are highly concordant. This contrasts classical case vs. control setups that focus on a specific and most often invasive condition, for example a disease phenotype, such that drastic changes in the entire blood-borne miRNome dominate the variance in expression. Regarding miR-532-5p as a candidate marker for training performance outcome after carbohydrate uptake, an independent study by Cui et al. found that miR-532-5p was de-regulated in participants conducting muscular strength endurance training, and it showed a negative correlation with the insulin-like growth factor-1 [34]. Moreover, several earlier publications investigated the prominent role of miR-532-5p in the context of adult obesity and diabetes type 2, in particular insulin resistance [35,36,37,38]. Interestingly, most of these studies have in common that miR-532-5p is down-regulated in patients suffering from obesity or diabetes. Setting these reports into context, our findings imply that miR-532-5p is up-regulated in individuals that exhibit a gain in training performance while consuming glucose before each training. This highlights a potential role of miR-532-5p in understanding homeostasis-related cell fitness as well as metabolic and systemic diseases, e.g., diabetes. It is important to note that the miR-532 precursor is located on the gonosomal X chromosome. Therefore, our results might be affected by a certain degree of gender bias due to a genetic imbalance of genomic locis. However, the gender distributions of our recommendation groups were not particularly skewed towards either female or male participants.

Taken together, our results imply a connection between important regulators in the non-coding transcriptome, i.e., circulating miRNAs, molecular factors, and phenotype in healthy human individuals, which might be characterized in a precise manner with the aid of larger validation studies.

## 4. Materials and Methods

### 4.1. Study Design

Our findings are based on a randomized cross-over study in 23 healthy, previously untrained adults [17]. In brief, two eight-week training periods were separated by a wash-out period of equally eight weeks. Training consisted of 4 times 45 min running/walking at 70% of heart rate reserve per week. Compliance was supervised and documented by supervised training sessions and read-out of heart rate monitors. Each participant received 50 g of glucose dissolved in 200 mL of water 15 min before every training session of one of the two training periods (N1: glucose during second training period; N2: glucose during first training period; N1 = 13, N2 = 10 consisting of fN1 = 6, mN1 = 7, and of fN2 = 4 females, mN2 = 6 males, respectively). VO_2_ max was determined by an exhaustive, ramp-shaped exercise test with gas exchange measurements before and after each period. Blood was collected before the exercise tests. The original study design, methods and protocols are in accordance with the Declaration of Helsinki and approved by the local ethics committee (Ärztekammer des Saarlandes, Saarbrücken, Germany; approval number: 148/10). Written informed consent was obtained from all participants before exercise testing. This trial was registered at clinicaltrials.gov as NCT02297646.

### 4.2. Blood Sampling and Rna Extraction

Whole blood was collected in PAXGene Blood RNA Tubes (BD Biosciences, San Jose, CA, USA) from the 3–4th quarters of 2012 through the 1st quarter of 2013 and frozen at −20 °C until RNA isolation. Total RNA including small RNAs were isolated during October and November in 2017 using PAXGene Blood miRNA kit (Qiagen, Hilden, Germany) according to manufacturers recommendations. RNA quantity and quality was checked with Nanodrop 2000 (Thermo Fisher Scientific, Waltham, MA, USA) and Bioanalyzer RNA 6000 Nano Kit (Agilent, Santa Clara, CA, USA), respectively. Isolated RNA was kept frozen at −80 °C and thawed before array preparation in December 2018 through January 2019.

### 4.3. Microarray Experiments

MiRNA profile was measured with Agilent Human miRNA microarray (miRBase v21, Santa Clara, CA, USA) according to the manufacturers protocol. In short, 100 ng total RNA was dephosphorylated and 3’ labelled with 3-pCp. Labelled RNA was hybridized to the array for 20 h at 55 °C an 20 rpm. Slides were washed and air-dried and subsequently scanned with Agilent Microarray Scanner (G2505C, Santa Clara, CA, USA) with 3 μm resolution in double-path mode. Signals were extracted using Agilent Feature extraction software (v10.10.1.1).

### 4.4. Statistical Analysis

Raw microarray data resulting from the Agilent array-scanner was parsed into a raw expression table and microRNA expression levels for the samples under consideration were quantile normalized and log${}_{2}$ transformed. We required a microRNA to be detected in at least 50% of the samples in order to be considered for subsequent analysis. Then, we discarded all microRNAs for which the 3rd quartile of log${}_{2}$ transformed expression values was below 3.5. Using sample Spearman correlation analysis we identified one outlier (Sample no. 26, mean(rho)≈0.76) with a mean pairwise correlation less than the set threshold of 85%, which was removed from the dataset before performing any tests. All data analysis was performed using the statistical programming language R v3.5.3. Expression and correlation heatmaps were created using the package pheatmap v1.10.12. All other main and supplementary figures were generated using the packages ggplot2 v2.2.1, ggfortify v0.4.6, ggsci v2.9, cowplot v0.9.4, grid v3.5.3, gridExtra v2.3, and Microsoft Powerpoint v16.28. Common data manipulations such as merging, filtering, and sorting of tables were accomplished with data.table v1.12.2, reshape2 v1.4.3, dplyr v0.8.1, stringr v1.4.0, tidyr v0.8.3, purrr v0.3.2, and tibble v2.1.1. Principal component analysis was taken out using the *prcomp* function, in addition to the Principal Variants Component Analysis that was taken from the package pvca v1.20.0, having fixed the percentage of variance explained threshold at 90%. The t-SNE analysis was done with Rtsne v0.15 using the following parameters: dims: 2, initial_dims: 50, perplexity: 20, theta: 0.0, check_duplicates: TRUE, pca: FALSE, normalize: FALSE and keeping the rest at default, while the UMAP analysis was performed with umap v0.2.2.0 and the parameters: method: ‘naive’, n_neighbors: 20, n_components: 2, metric: ‘euclidean’, n_epochs: 500, and min_dist: 0.1, also keeping all other parameters at default. *p*-Values are adjusted with FDR<0.05 unless stated otherwise in the main text. Output of R builtin base functions such as t.test was transformed to data.frames using the broom package v0.5.2. Machine learning procedures were performed with caret v6.0-84. For the microRNA-VO${}_{2}$ max regression model we used repeated cross-validation with 10 repeats and 6 folds each. As data model glmnet with a tunelength of 50 was applied to find the best hyperparameters. Variable feature importance is defined as the absolute coefficient value resulting from the trained linear regression model. To compute the correlation between miRNA expression and VO${}_{2}$ max absolute measurements are used, while the CHO recommendation group assignment is based on ΔVO${}_{2}$ max values between adjacent timepoints.

### 4.5. Data Availability and Accession Codes

Microarray data is available through NCBI’s Gene Expression Omnibus (GEO) using the Accession ID GSE133910.

## 5. Conclusions

Further and independent cross-over studies are required in order to prove the role of the miRNA profiles and the candidate marker miR-532-5p as described in this study. A more encompassing characterization of the human blood-borne miRNome through next-generation-sequencing might reveal novel hypotheses and genetic variants that could be related to an endurance-training driven change in VO${}_{2}$ max.

## Figures and Tables

**Table 1:** Significantly de-regulated miRNAs that exhibit an absolute log${}_{2}$ fold change ≥1 and a *p*-value less than 0.05. Results for the three setups correspond to the volcano plots shown in Figure 3a,c,e.

Direction	Setup 1	Setup 2	Setup 3
Up-regulated			
	let-7a-5p	let-7a-5p	let-7f-5p
	let-7f-5p	let-7f-5p	let-7g-5p
	let-7g-5p	let-7g-5p	miR-17-3p
	miR-17-3p	miR-15a-5p	miR-26b-5p
	miR-20a-5p	miR-16-5p	miR-98-5p
	miR-20b-5p	miR-17-3p	miR-144-3p
	miR-26b-5p	miR-17-5p	miR-144-5p
	miR-98-5p	miR-18a-5p	miR-199a-3p
	miR-126-3p	miR-18b-5p	miR-1246
	miR-144-3p	miR-20a-5p	
	miR-144-5p	miR-20b-5p	
	miR-195-5p	miR-26b-5p	
	miR-199a-3p	miR-93-5p	
		miR-98-5p	
		miR-126-3p	
		miR-144-3p	
		miR-195-5p	
		miR-199a-3p	
		miR-374a-5p	
		miR-374b-5p	
		miR-454-3p	
		miR-7107-5p	
Down-regulated			
	miR-30b-5p	miR-16-2-3p	miR-30b-5p
	miR-30c-5p	miR-30a-5p	miR-30c-5p
	miR-199a-5p	miR-30b-5p	miR-192-5p
	miR-223-3p	miR-30c-5p	miR-199a-5p
	miR-326	miR-30d-5p	miR-223-3p
	miR-331-3p	miR-326	miR-340-3p
	miR-340-3p	miR-331-3p	miR-378a-5p
	miR-378a-5p	miR-378a-5p	miR-484
	miR-484	miR-484	miR-550a-3p
	miR-550a-3p	miR-550a-3p	miR-7977
	miR-7977		

**Table 2:** Mean and standard deviation of miR-532-5p expression in samples from the two glucose recommendation groups split by the four study timepoints ($E_{1}$, $A_{1}$, $E_{2}$, $A_{2}$). Samples from a non-treatment interval are marked with −Glucose. Conversely, + Glucose notes scores generated from treatment samples. *p*-Values were obtained with Welch two sample t-tests using samples from each recommendation group with the same pre- (Timepoint 1) or post-training (Timepoint 2) label in either of the two treatment intervals (− Glucose, + Glucose).

	Timepoint 1 ($E_{1}\vee E_{2}$)		Timepoint 2 ($A_{1}\vee A_{2}$)	
	Group 1 (+)	Group 2 (−)	Group 1 (+)	Group 2 (−)
− Glucose	μ≈6.409,σ≈0.231	μ≈6.221,σ≈0.312	μ≈6.727,σ≈0.233	μ≈6.356,σ≈0.396
	p=0.1538		p=0.02359 (*)	
+ Glucose	μ≈6.261,σ≈0.392	μ≈6.361,σ≈0.225	μ≈6.389,σ≈0.531	μ≈6.387,σ≈0.315
	p=0.5135		p=0.9941	

## Supplementary Materials

The following are available online at https://www.mdpi.com/2073-4409/8/9/1045/s1.

## Abbreviations

The following abbreviations are used in this manuscript:

VT1	Ventilatory threshold 1
VO${}_{2}$ max	Maximal aerobic capacity
miRNA	MicroRNA
miRNome	Set of expressed miRNAs in a cell-type or tissue
mRNA	Messenger-RNA
UTR	Untranslated region
cDNA	Complementary DNA
CHO	Carbohydrates
AMPK	AMP-activated protein kinase
ECG	Electrocardiography
CBC	Complete blood count
RBC	Red blood cell
BMI	Body mass index
PCA	Principal component analysis
PVCA	Principal variance component analysis
t-SNE	t-distributed stochastic neighbor embedding
UMAP	Uniform manifold approximation and projection
GEO	Gene expression omnibus
