# Supplementary material for: Systematic Assessment of Blood-Borne MicroRNAs Highlights Molecular Profiles of Endurance Sport and Carbohydrate Uptake
Source: Cells. 2019 Sep 6;8(9):1045. doi: 10.3390/cells8091045 (PMC6770460; doi:10.3390/cells8091045)
Supplement: Supplementary file 1 [file cells-08-01045-s001.zip › supplementary_data/supplementary_data.pdf]

Systematic assessment of blood-borne microRNAs highlights molecular profiles of endurance sport and carbohydrate uptake

Supplementary data

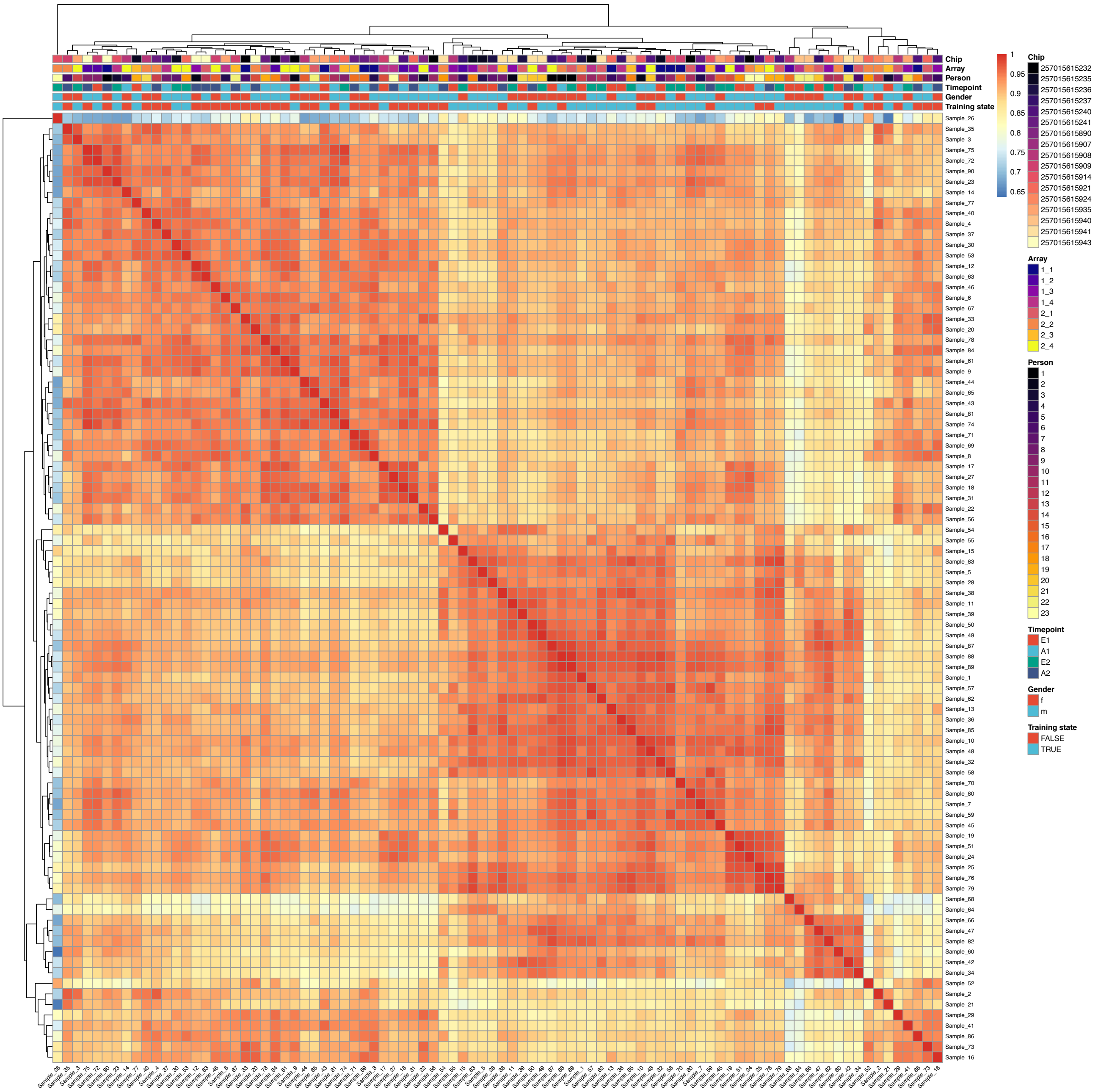

Figure S1: Sample to sample Spearman correlation matrix. Color coding according to correlation values where a higher correlation is shown in red, while lower values are shown in blue. Along each dimension a hierarchical clustering is plotted as a dendrogram. Color bars on top of the matrix encode for different annotation variables for each of the samples. The top and leftmost sample no. 200 was identified as an outlier.

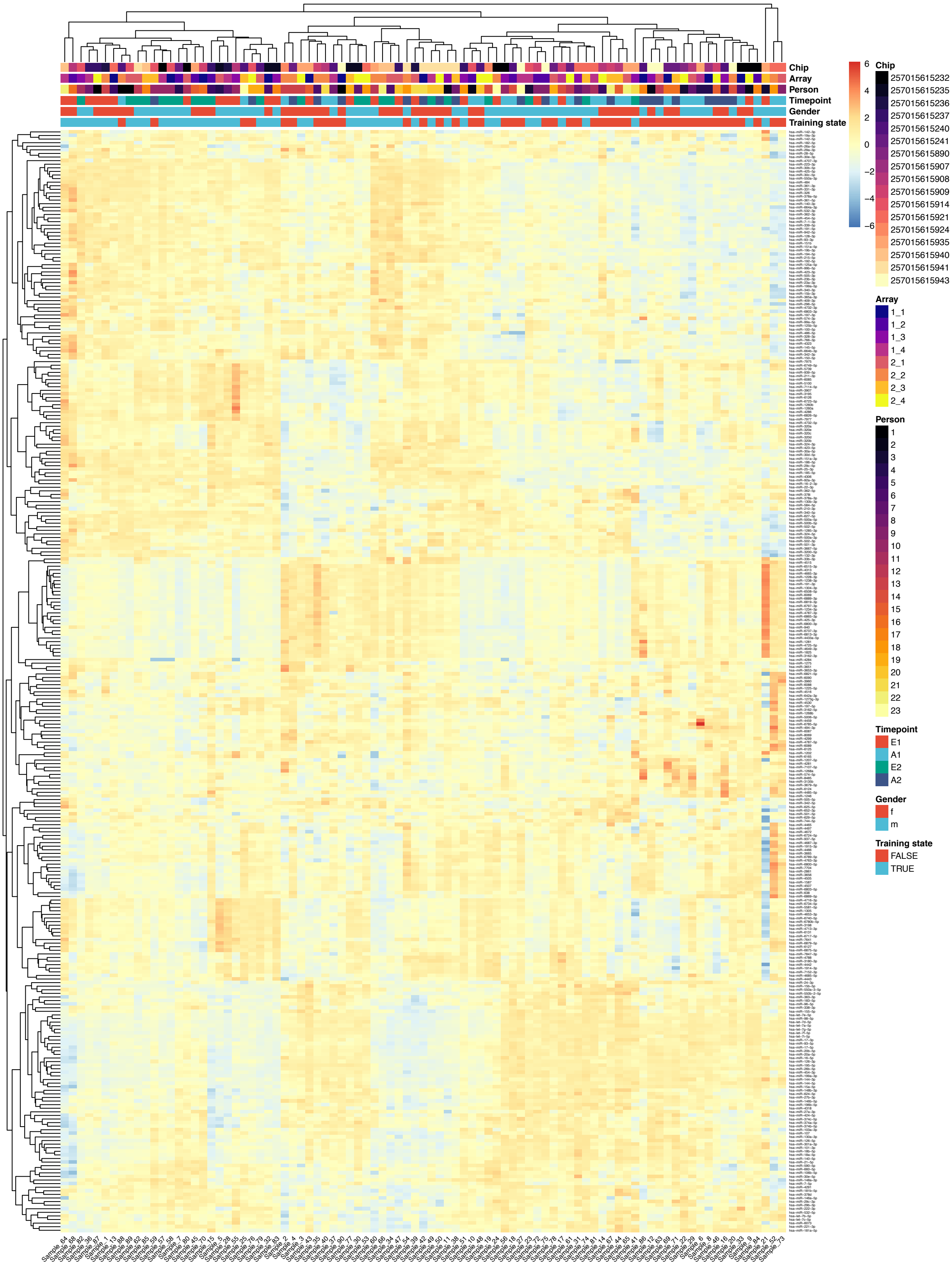

Figure S2: MiRNA to sample expression matrix using Z-scores. Higher expression values are color coded in red, while lower expression values are colored in blue. Along each dimension a hierarchical clustering is plotted as a dendrogram, i.e. for samples on top and miRNAs on the left of the heatmap. Color bars on top of the matrix encode for different annotation variables for each of the samples. Several clusters of miRNAs that show a similar expression profile can be identified form the expression matrix and the clustering.

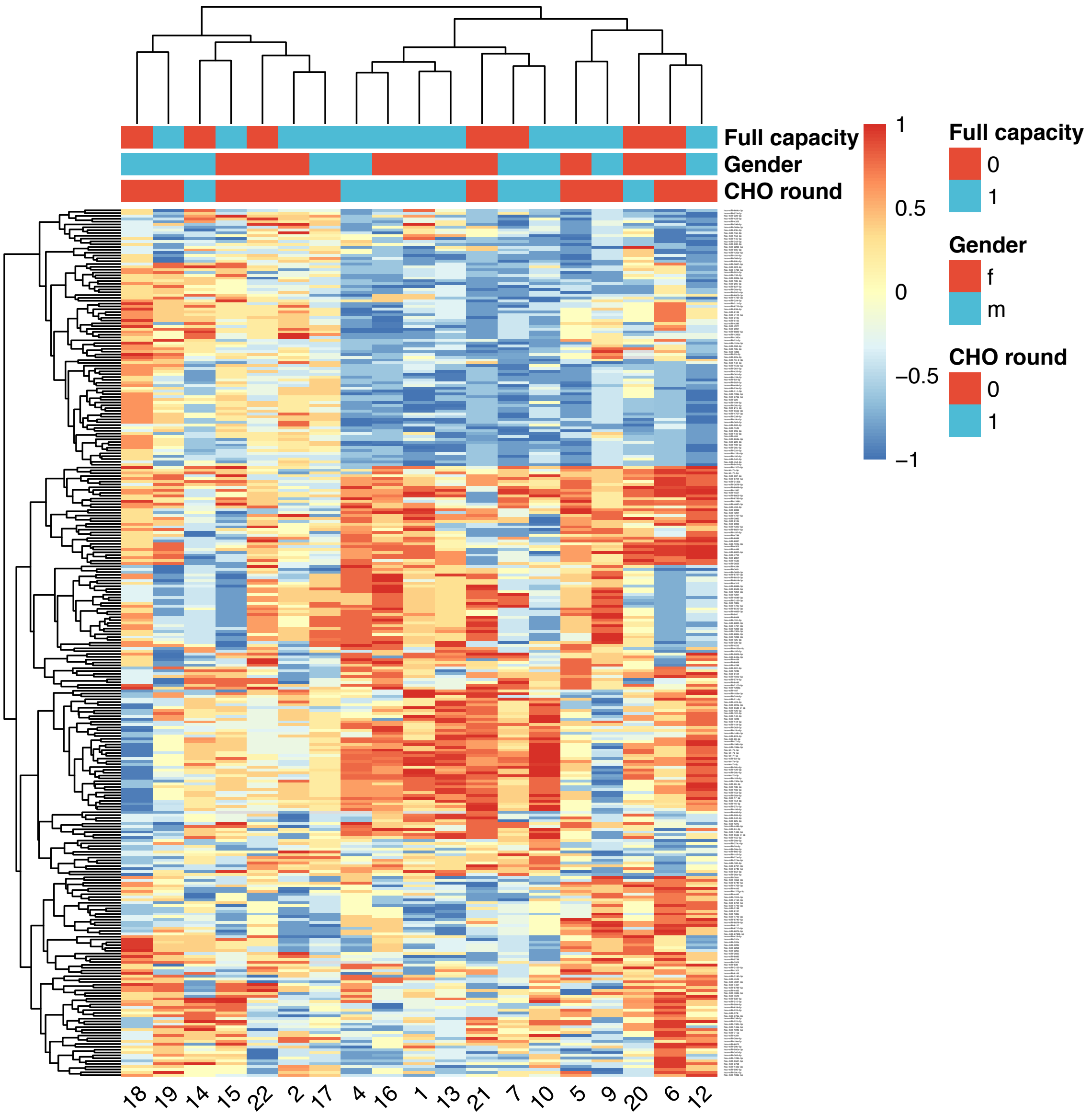

Figure S3: Correlation matrix between measured VO2 max levels and miRNA expression distributed over the study participants. A high positive correlation is colored in red, while a strong negative correlation is colored in blue. Along each dimension a hierarchical clustering is plotted as a dendrogram, i.e. for participants on top and miRNAs on the left of the heatmap. Color bars on top of the matrix encode for three binary annotation variables obtained for each of the participants. Similar to Supplementary Figure S2, several clusters of miRNAs that show comparable correlations with VO2 max levels can be identified.

Table S1: Study overview, anthropomorphic variables, and group assignments for each participant.

Table S2: Blood sample measurements as well as anthropomorphic and fitness parameters obtained from participants across each timepoint of the study.

Table S3: Aggregated measures and statistical test results for variables re-obtained at each of the four study timepoints.

Table S4: MiRNA coefficient loadings obtained with Principal Components Analysis. Higher values indicate features that explain more variance in the dimensions spanned by the Principal Components.

Table S5: MiRNA cluster assignments defined by cutting the hierarchical clustering dendrogram of expression values into six distinct sub-trees.

Table S6: MiRNA variable importance assigned by performing linear regression on the VO2 max levels using expression vectors as features. A higher variable importance indicates a stronger approximated effect on VO2 max levels. In particular, correlated miRNAs can share a high coefficient value.
